# Comparative proteoinformatics revealed the essentials of SDS impact on HaCaT keratinocytes

**DOI:** 10.1038/s41598-022-25934-4

**Published:** 2022-12-12

**Authors:** Timur Shkrigunov, Yulia Kisrieva, Natalia Samenkova, Olesya Larina, Victor Zgoda, Alexander Rusanov, Daniil Romashin, Natalia Luzgina, Irina Karuzina, Andrey Lisitsa, Natalia Petushkova

**Affiliations:** 1 Center of Scientific and Practical Education, Institute of Biomedical Chemistry, Moscow, Russia 119121; 2 Laboratory of Microsomal Oxidation, Institute of Biomedical Chemistry, Moscow, Russia 119121; 3 Laboratory of Systems Biology, Institute of Biomedical Chemistry, Moscow, Russia 119121; 4 Laboratory of Precision BioSystems, Institute of Biomedical Chemistry, Moscow, Russia 119121

**Keywords:** Biochemistry, Proteomics, Bioinformatics, Proteomic analysis, Toxicology, Data processing

## Abstract

There is no direct evidence supporting that SDS is a carcinogen, so to investigate this fact, we used HaCaT keratinocytes as a model of human epidermal cells. To reveal the candidate proteins and/or pathways characterizing the SDS impact on HaCaT, we proposed comparative proteoinformatics pipeline. For protein extraction, the performance of two sample preparation protocols was assessed: 0.2% SDS-based solubilization combined with the 1DE-gel concentration (Protocol 1) and osmotic shock (Protocol 2). As a result, in SDS-exposed HaCaT cells, Protocol 1 revealed 54 differentially expressed proteins (DEPs) involved in the disease of cellular proliferation (DOID:14566), whereas Protocol 2 found 45 DEPs of the same disease ID. The ‘skin cancer’ term was a single significant COSMIC term for Protocol 1 DEPs, including those involved in double-strand break repair pathway (BIR, GO:0000727). Considerable upregulation of BIR-associated proteins MCM3, MCM6, and MCM7 was detected. The eightfold increase in MCM6 level was verified by reverse transcription qPCR. Thus, Protocol 1 demonstrated high effectiveness in terms of the total number and sensitivity of MS identifications in HaCaT cell line proteomic analysis. The utility of Protocol 1 was confirmed by the revealed upregulation of cancer-associated MCM6 in HaCaT keratinocytes induced by non-toxic concentration of SDS. Data are available via ProteomeXchange with identifier PXD035202.

## Introduction

One vital function of the skin is to form an effective barrier between the organism and the environment. The outer layer of skin—the epidermis—comprises stratified squamous epithelial cells. Keratinocytes constitute a major part of the human skin epidermis (accounting for approximately 90% of all epidermal cells) and form a barrier against the damage of xenobiotics, heat, UV radiation, pathogenic bacteria, fungi, parasites, and viruses. Keratinocytes produce numerous antimicrobial molecules, further contributing to a robust protective blockade. Besides that, keratinocytes act as immunomodulators after skin injuries.

HaCaT cells are keratinocytes that have been spontaneously immortalized in vitro from a histologically normal human epithelial cell line from adult skin that still has comprehensive epidermal differentiation capacity. HaCaT cells respond to external signals similarly to normal keratinocytes despite the altered and unlimited growth potential. Under appropriate conditions, all major epidermal differentiation products (specific keratins, involucrin, filaggrin) are expressed apparently in the right sequence^1^. HaCaT cell line is a readily available cell culture model that can be used as an alternative to primary or low-passaged normal keratinocytes for testing and detection of growth and differentiation factors, as well as the introduction of exogenous genes or subgenomic regulatory elements. Thereunder, HaCaT cells have been widely used as a cellular model for studying in vitro cytotoxicity caused by various materials, chemicals, drugs, and other substances. For the purpose of assessing the cytotoxic effects of surfactants, sodium dodecyl sulfate (SDS) is widely used as a reference compound which causes dose-dependent HaCaT cells’ viability decrease proportional to the exposure duration^2,3^. Moreover, SDS is the most widely used anionic alkyl sulfate surfactant, which makes it important in hundreds of household and industrial cleaning products, personal care products and cosmetics. Its environmental occurrence arises mainly from its presence in complex domestic and industrial effluents but also due to the direct release in some applications (e.g., oil dispersants and pesticides). SDS can enhance the absorption of chemicals through the skin, gastrointestinal mucosa, and other mucous membranes. Thus, it is used in transepidermal, nasal, and ocular drug delivery systems and to enhance the intestinal absorption of poorly absorbed drugs^4^. Direct contact with SDS (≤ 20%) may cause moderate inflammation, irritation of the skin, eyes, mouth, lungs, and this surfactant may slowly build up in skin cells over long-term use. Although there is no direct evidence supporting that SDS is a carcinogen^5^, we have previously demonstrated that SDS exposure resulted in the upregulation of proteins that control glycolysis^6^. There was a correlation between enhanced glycolytic ATP production and tumor malignancy^7^. The development of cutaneous carcinogenesis is a highly complex process mediated by numerous proteins. Its investigation requires significant consideration during the proteoinformatics workflow, particularly in the sample preparation (including extraction, digestion, and so on), mass spectrometry analysis, and data processing, to increase the incidence of revealing the key proteins and/or pathways^8^.

The efficient extraction of proteins of interest from cells is not always simple. The choice of the method of cell destruction depends on the type of cells, the amount and the physical properties of the extracted proteins. Several methods are commonly used for cell lysis, including mechanical destruction, osmotic shock, ultrasound, multiple freeze–thaw cycles, and homogenization^9^. In order to facilitate the extraction of labile proteins, osmotic shock is widely used as a cell lysis method^10,11^. However, compared to various cell disruption methods, the osmotic shock alone is relatively inefficient in cell disruption with low protein extraction. Detergent-based cell lysis is considered the most optimal way to destroy cell membranes^12^. Detergents break the lipid barrier surrounding cells by solubilizing proteins and disrupting lipid-lipid, protein–protein, and protein-lipid interactions. Nevertheless, similar to osmotic shock, it is often used in conjunction with homogenization, mechanical grinding, and high-frequency sound waves (sonication) when preparing protein samples to achieve complete cell disruption^13^. On the other hand, the sonication of the lysate on ice for 15–30 s disrupts cellular components and genomic DNA, preventing them from interfering with further sample preparation^14^.

SDS is believed to be a frequently used detergent for cell lysis (release of soluble proteins) and membrane protein solubilization in proteomic analysis of various human tissues and cells^15,16^. SDS is a strong lysis agent that works with most cell types^17^. This detergent binds to proteins via ionic and hydrophobic interactions and solubilizes ones with diverse physical properties by modifying their secondary and tertiary structures. However, SDS is incompatible with protease activity and mass spectrometry, so its use in proteomic analysis is limited^18^. Since detergent removal is essential for the subsequent in-depth proteome profiling, this stage is mandatory in the sample preparation workflow. SDS can be removed using various techniques, including acetone precipitation, strong cation exchange, protein and peptide level purification with Pierce detergent removal cartridges, FASP II, and others^19^. One of the best ways of SDS removal is gel electrophoresis, during which the proteins will be gel-bound, perfectly denatured and ready for in-gel digestion^20^. Our group recently developed a sensitive SDS cleanup protocol called 1DE-gel concentration that meets all of the criteria we believe are essential for an optimal sample processing approach^21^. The 1DE-gel concentration is based on using a polyacrylamide stacking gel (4%T) without protein separation before in-gel trypsin digestion. Unlike the classic SDS-PAGE, which produces many protein bands separated by molecular weight, a single band is obtained after the 1DE-gel concentration. The obtained single protein band includes almost all the proteins of the examined tissue after SDS solubilization. Such a way of removing SDS showed high-quality results in analyzing proteins in SDS extracts of human chorionic villi^21^.

The aim of the present study was to select candidate proteins and/or pathways representing the SDS impact on HaCaT keratinocytes using comparative proteoinformatics. Implementing the in-depth proteomic characterization of HaCaT cells, we assessed the performance of two sample preparation procedures: 0.2% SDS-based solubilization + 1DE-gel concentration procedure (Protocol 1) and protein extraction by osmotic shock (Protocol 2).

## Results and discussion

### Characterization of HaCaT cell proteome with different sample preparation protocols

Sample preparation is a key stage in a complete proteomic experiment of tissues and cells, as it can significantly impact the sensitivity of the downstream analysis. The principal challenge during sample preparation is to extract all proteins in a way that enables efficient digestion and is compatible with subsequent mass spectrometric analysis^22^. Cell lysis with detergents is frequently utilized with cultured mammalian cells. We used an SDS-containing buffer for HaCaT cell lysis because SDS provides effective solubilization and dissociation of most lipid-protein and protein–protein interactions, thereby separating proteins^23^. However, SDS is an exceptionally strong detergent^12,17^; it is likely to denature all proteins it comes in contact with. Therefore, in this study, we investigated the effect of two sample preparation approaches on the quality of HaCaT cell proteome analysis. Protocol 1 included 0.2% SDS-based protein solubilization in conjunction with 1DE-gel concentration for SDS removal. Protein extraction by osmotic shock was the framework of sample preparation Protocol 2.

**Figure 1:**
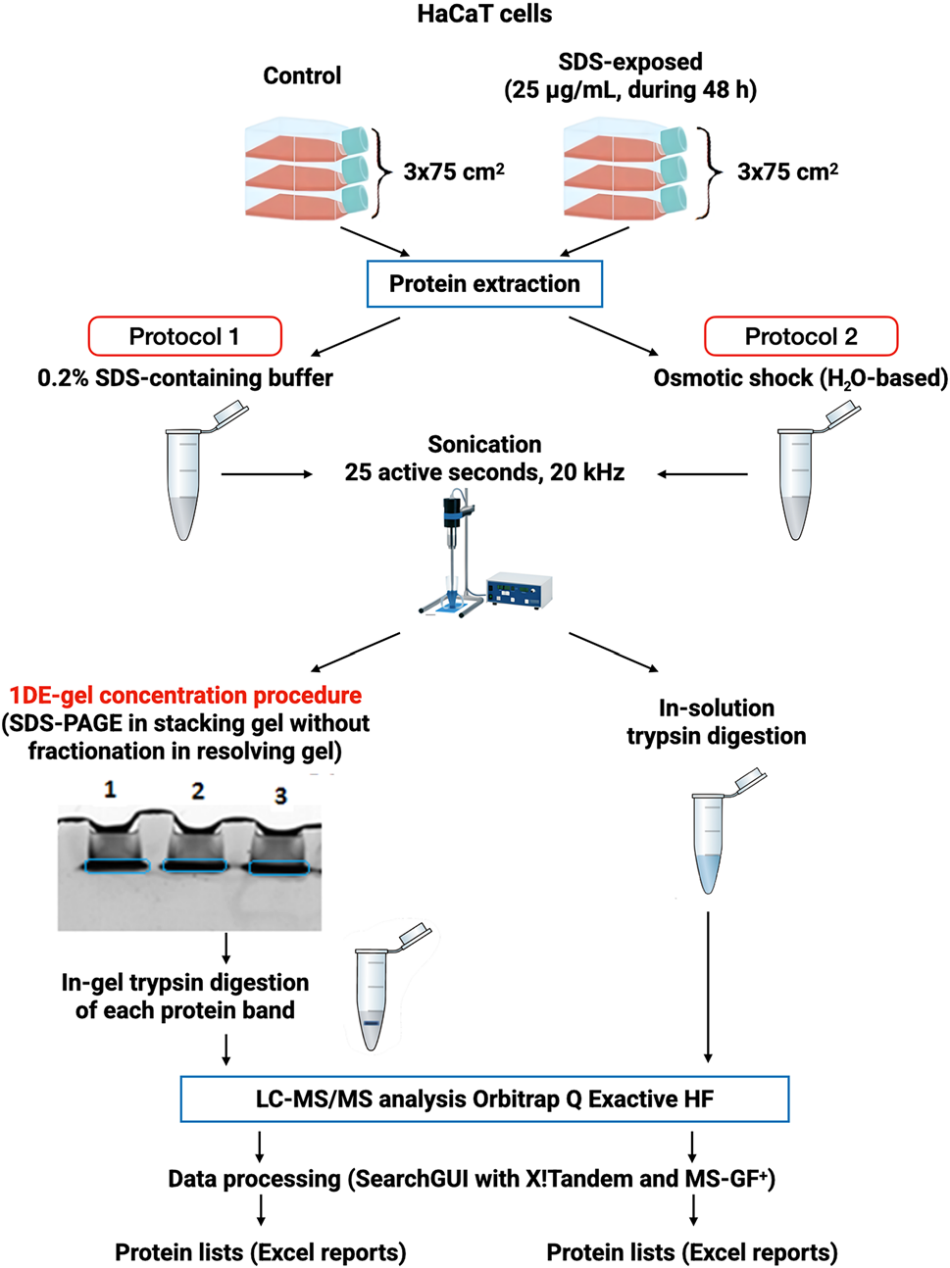
The experimental design used in the proteomic study of HaCaT keratinocytes. After growing to ~ 60–70% confluence, the cultured cells were divided into two groups, one was control (fresh medium was added), and another was exposed to SDS in the non-toxic dose (25 μg/mL) during 48 h. Protocol 1—HaCaT cells were solubilized in 0.2% SDS-based solution and sonicated. The obtained protein extracts were deposited onto polyacrylamide stacking gel (4%T, in triplicate, 50 μg of protein per gel run). Electrophoresis (50 V, 20 min) was terminated before the migration of Bromophenol blue in the resolving gel. The single protein band was excised from gel holistically and digested with trypsin. Protocol 2—HaCaT cells were mixed with cold deionized water and sonicated. Then, the in-solution trypsin digestion was performed. In both protocols, the resulting mixture of peptides was extracted for LC-MS/MS analysis. The spectra were processed with the SearchGUI platform utilizing X!Tandem and MS-GF + search algorithms. Processing parameters are given in the Methods section.

A schematic representation of the HaCaT proteomic research strategy is shown in Fig. 1. The pooling method was used for three biological replicates (cell culture flasks) of each keratinocyte type (control and SDS-exposed). In both sample preparation procedures, sonication was utilized for a more complete release of the cell protein content. The additional SDS cleanup step before trypsin digestion was used in Protocol 1—1DE-gel concentration in an upper stacking 4% polyacrylamide SDS-PAGE gel^21^. 1DE-gels represent a simple, widely available technique that can either fractionate complex proteomes or rapidly clean up low microgram samples with minimal losses^24^. Our previous studies on the human chorionic villi lysates have shown reproducible and highly sensitive protein identification with 1DE-gel concentration. It turned out that the run time in the stacking gel depends on the type of biological samples. It was previously shown that this time was 45–50 min for the human chorionic villi protein mixture^21^, whereas the run time for HaCaT cells was only 15–20 min.

Protocol 1 identified a total of 2989 proteins from the HaCaT cell line, of which 2579 proteins were in control and 2520 proteins in the SDS-treated samples. In such a way, the 1DE-gel concentration reduces the time consumption and the laboriousness of the sample preparation procedure due to the skipping of proteins fractionation in a resolving gel (12%T) stage involved in traditional SDS-PAGE. The resulting single protein band includes almost all the proteins of the examined tissue after SDS solubilization, and the number of samples for subsequent enzymatic digestion and mass spectrometric analysis may be decreased. With all this, there was no reduction in the output of protein identifications looking up to classical SDS-PAGE^25^.

The detergent concentration is crucial for higher yield, as well as for improving the stability and integrity of proteins. The concentration of 1–2% SDS is widely used for protein extraction from cultured cell lines^26^. Using the 2% SDS concentration in solubilizing buffer led to a decrease in MS identifications (unpublished data). In such a way, it is essential to choose the proper concentration of detergent for each type of biological sample.

Osmotic shock is the technique of weakening cells caused by lysis, which is brought about by increased internal pressure as buffer rapidly enters cells when cells are subjected to high osmotic pressure followed by sudden dilution^27^. Compared to other cell disruption methods, osmotic shock is typically characterized by low protein extraction and relatively inefficient cell disruption^28^. Indeed, the total number of proteins detected with sample preparation Protocol 2 was 1448, with 1222 ones identified in SDS-treated cells and 1084 in control.

To define the intersections between protein content obtained with two sample preparation protocols (1DE-gel concentration/osmotic shock) on two types of HaCaT cells (with/without SDS treatment), we performed SearchGUI processing by joining technical repeats within each dataset. As a result, four protein datasets (Excel reports) were used for further analysis. One challenge of any proteomic experiment is keratin contamination. It may arise from dead cells, dust, surfaces, lab coats, latex gloves, etc. We analyzed initial reports on common keratin contaminants’^29^ presence and found KRT19, KRT7, KRT16, KRT5, and KRT15 but our subsequently proposed comparative proteoinformatics pipeline did not pass them into the scope of data processing. In order to avoid false-positive findings, subsequent processing was performed within protein identifications by ≥ 2 unique validated peptides. The records on proteins (including the number of validated unique peptides per protein, score, sequence coverage and spectrum counting NSAF) identified in HaCaT cells by each of the two sample preparation protocols in two origins of keratinocytes are presented in Supplementary Tables S1, S2, S3 and S4.

**Figure 2:**
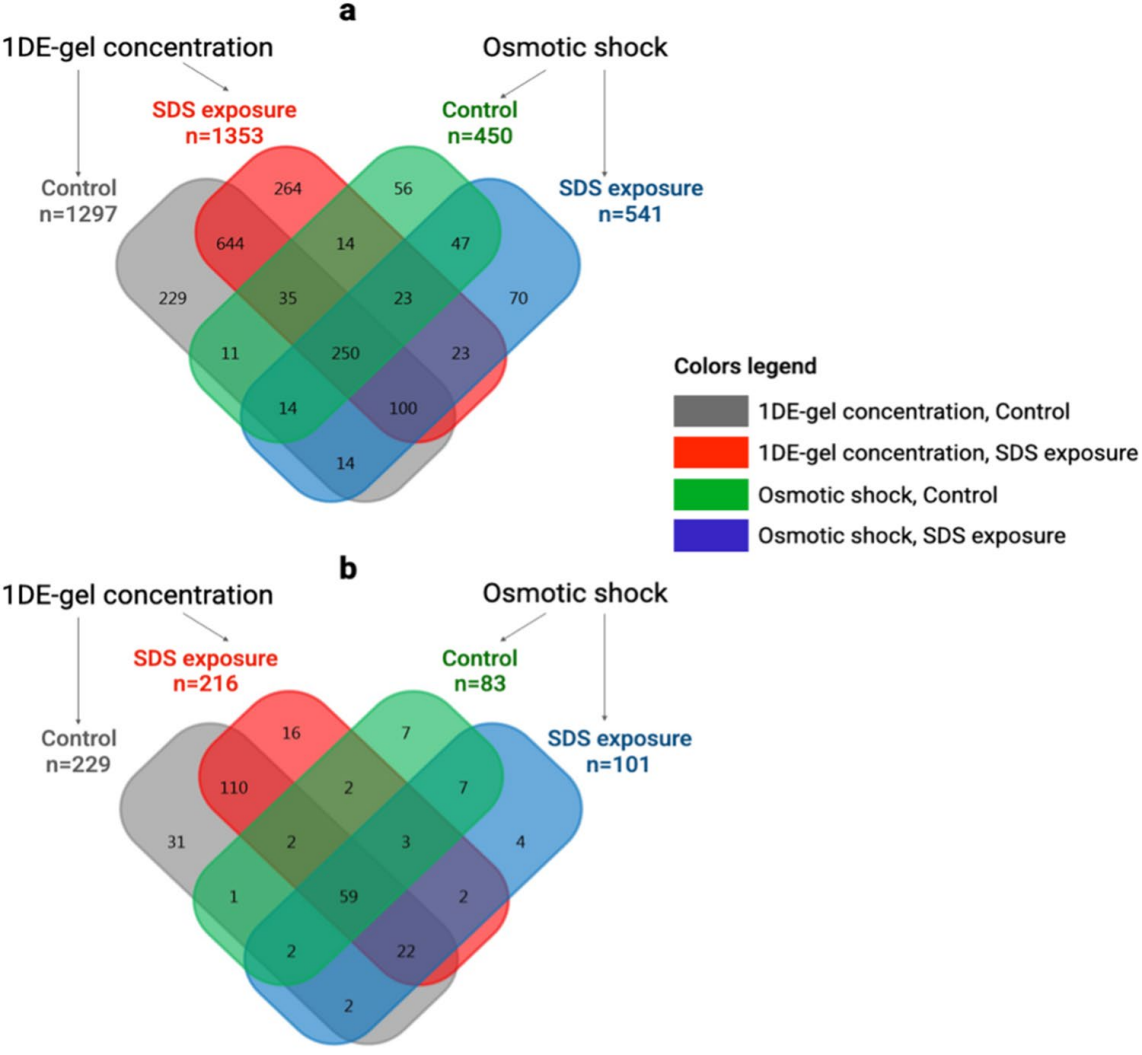
Unique and shared proteins identified by ≥ 2 validated unique peptides in control and SDS-exposed HaCaT keratinocytes treated with two sample preparation methods: 0.2% SDS solubilization + 1DE-gel concentration and H_2_O-based osmotic shock. Venn diagrams on (**a**) total numbers of proteins (**b**) skin-related proteins (according to the Human Protein Atlas database, v. 20062022, https://www.proteinatlas.org/search/skin) are shown.

Comparing total protein lists (identifications by ≥ 2 unique validated peptides), there were 250 shared proteins (Fig. 2a) related to 4 datasets (Fig. 1, colors legend: HaCaT cells with/without SDS exposure processed with sample preparation Protocol 1 of 0.2% SDS solubilization + 1DE-gel concentration and Protocol 2 based on H_2_O-based osmotic shock). The number of uniquely identified proteins was highest for both control and SDS-exposed keratinocytes prepared with Protocol 1.

In the next step, skin-related proteins were selected (according to the Human Protein Atlas database, v. 20062022, https://www.proteinatlas.org/search/skin). We found 59 skin-related proteins related to all four datasets (Fig. 2b). Overall, we identified 253 and 113 skin-related proteins by utilizing Protocols 1 and 2, respectively. Despite the differences in these values, they both match approximately 16% of all protein identifications within each tested sample preparation method. The number of skin-related uniquely identified proteins was also the highest for both control (31 vs. 7) and SDS-exposed (16 vs. 4) keratinocytes treated with sample preparation Protocol 1 compared to Protocol 2.

**Figure 3:**
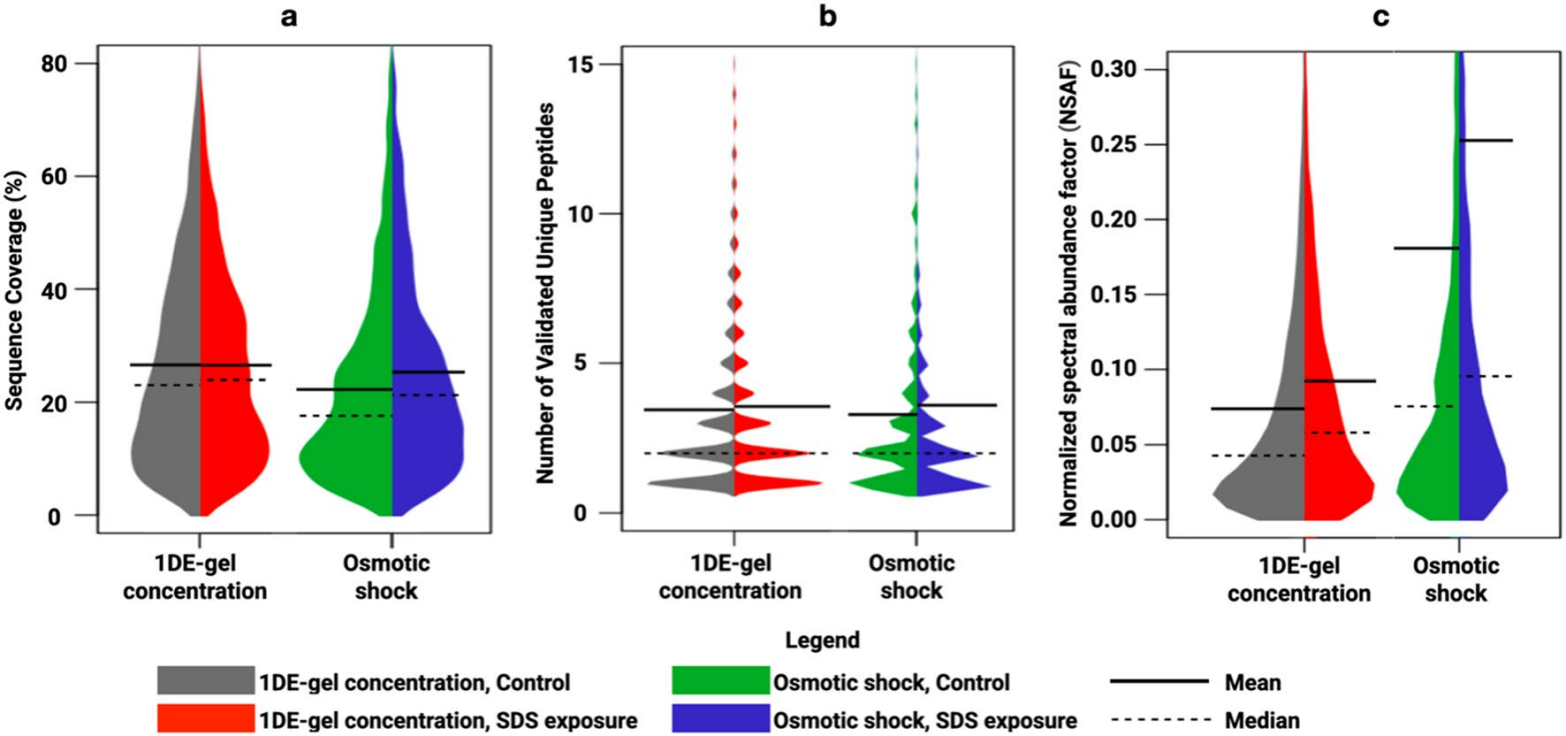
Qualitative and quantitative comparison of the identified proteins extracted from control and SDS-exposed HaCaT keratinocytes using 0.2% SDS lysis + 1DE-gel concentration approach and osmotic shock procedure. Violin plots give a glance at the statistics of (**a**) validated sequence coverage percentages on proteins; (**b**) the number of validated unique peptides identifying the proteins; (**c**) spectrum counting NSAF values scatter. Solid lines indicate the means, and dashed lines—the median values.

We used violin plots^30^ to visualize a distribution of several additional protein identification parameters depending on the sample preparation procedure and the origin of the HaCaT cells (with/without SDS treatment). The violin plot is a hybrid of a box plot and a kernel density plot, which shows the scatter of values. Sample preparation Protocol 1 did not result in discernible differences in mean and median sequence coverage percent values in control and SDS-exposed HaCaT cells (Fig. 3a). However, the average and median values for this parameter were slightly lower for both cell origins treated with Protocol 2. Contemporaneously, neither Protocol 1 nor Protocol 2 impacted the mean or the median number of validated peptides per protein (Fig. 3b). Conversely, SDS treatment resulted in a rise in mean and median values of normalized spectral abundance factor (NSAF)^31^ per protein, suggesting protein upregulation (Fig. 3c). As can be seen from Fig. 3, there is only a slight difference between the protein identification parameters distribution shapes.

Despite the loss in the number of protein identifications when using Protocol 2, the highest median and mean NSAF values were observed, which may indicate lower damage to intracellular proteins throughout osmotic cell lysis. Strong ionic detergent SDS helps to solubilize membrane proteins and lipids, thereby causing the cell to lyse and release its contents. This was confirmed in terms of the quality of the HaCaT proteome; samples prepared by Protocol 1 exhibited higher proteome coverage. However, this harsh detergent can often damage or destroy the contents of the cell (proteins). Nevertheless, a remarkable agreement in the sequence coverage and the number of unique peptides per protein was obtained between the sample preparation procedures compared in this work, confirming the effectiveness of SDS lysis during Protocol 1 in the MS analysis of the HaCaT keratinocyte proteome.

### Verification of differences in the number of identified proteins

To verify the increase in the number of Protocol 1 identifications, we compared the numbers of (I) proteins identified in the control and SDS-exposed HaCaT cells and (II) proteins detected by applying sample preparation Protocols 1 and 2. For this purpose, each technical repeat (MGF file) was processed independently to provide the analyzing numbers of identifications. The number of technical repeats in the case of Protocol 1 was three, both for control and SDS-exposed cells. There were four and five technical repeats in Protocol 2, for control and SDS exposure, respectively. Generally, for control and SDS-exposed cells treated with Protocol 1, the numbers of identified proteins were 782 ± 337 and 1047 ± 57 (mean ± standard deviation), respectively. Referring to the results of Protocol 2, for cells of control and SDS exposition groups, the numbers of identifications were 275 ± 92 and 318 ± 86 (mean ± standard deviation), respectively. In such a way, either joint (Fig. 2a) or replicates independent processing indicated that Protocol 1 exhibited a greater number of identified proteins, in comparison with a similar analysis of data obtained by Protocol 2.

In order to define the significance level of differences in identifications numbers, the Student’s test (a parametric unpaired hypothesis test on the means of two samples) was chosen as a tool for comparison because it can be applied for extremely small (N ≤ 5) unequal samples^32^. (1) Comparison did not reveal any statistical differences between the numbers of proteins identified in control and SDS-exposed cells (for Protocol 1: t = 1.36, P = 0.25, n = 3 and 3; for Protocol 2: t = 1.05, P = 0.33, n = 4 and 5; at alpha level 0.05). (2) However, there were statistical differences between identifications of control cells proteomes accessed via two protocols (t = 2.95, *P* = 0.03, n = 3 and 4; at alpha level 0.05). As well, numbers of proteins in reports obtained with Protocols 1 and 2 on SDS-exposed cells showed significant differences (t = 12.51, *P* < 1.6e−5, n = 3 and 5; at alpha level 0.05). Similar results were obtained for skin-related proteins (data not shown).

Thus, in our experimental design, the sample preparation Protocol 1 based on 0.2% SDS lysis + 1DE-gel concentration led to deeper coverage of the HaCaT cells proteome. The increased MS-based protein detection sensitivity of Protocol 1 applied to cultured cells is in concordance with the results obtained earlier for human chorionic villi treated with the same sample preparation protocol. In chorionic villi, the larger total number of proteins made it possible to identify low-abundance proteins, some of which had not been previously detected via mass spectrometry^21^. Furthermore, covering a larger part of the HaCaT proteome with Protocol 1 might be useful to reveal key proteins and/or pathways related to SDS impact on human skin keratinocytes.

### GO enrichment analysis

**Figure 4:**
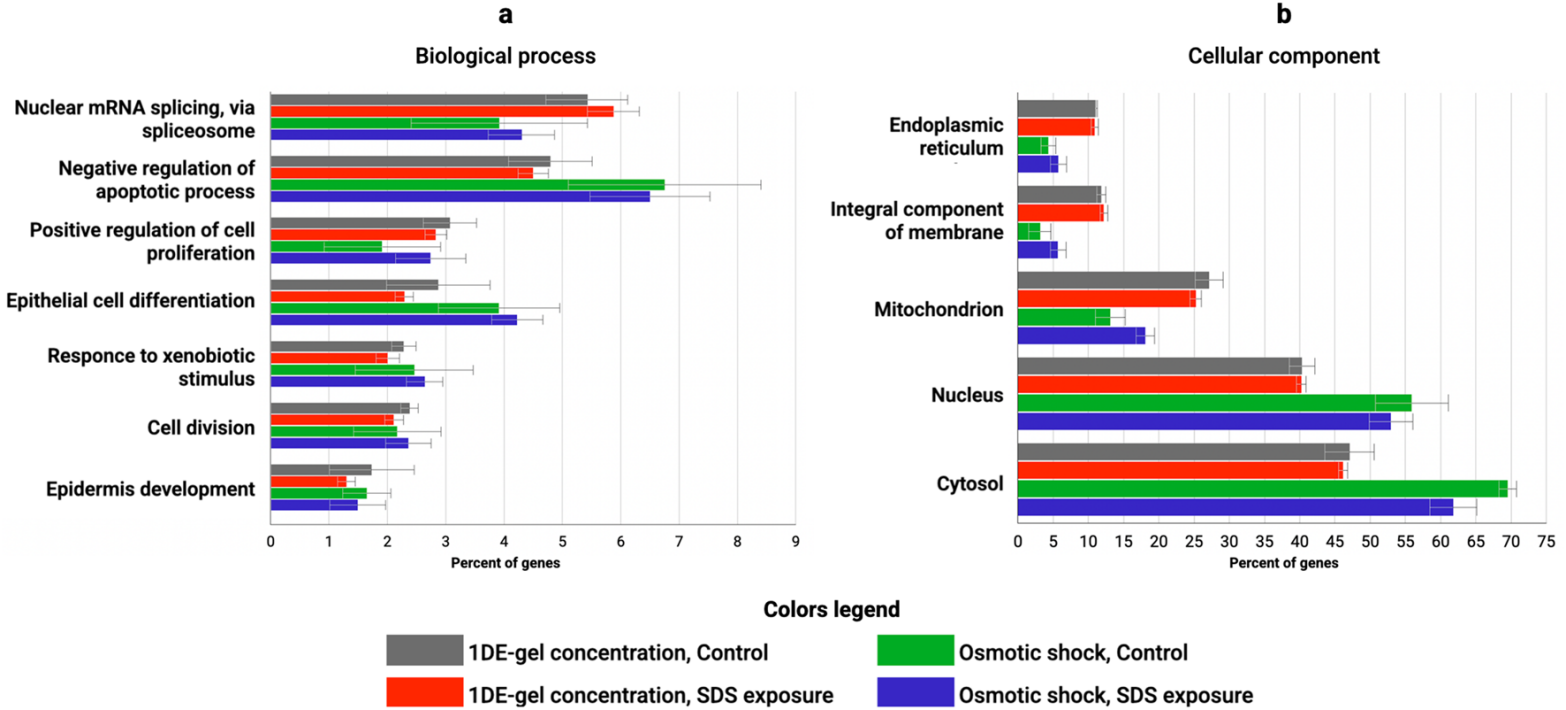
GO enrichment analysis of the proteins identified by ≥ 2 unique validated peptides in (**a**) biological process and (**b**) cellular component. Error bars represent the standard deviation.

GO enrichment analysis was performed to identify cellular components and perturbed biological processes between all biological samples (untreated keratinocytes and cells cultured under SDS exposure, cells processed with Protocol 1 and Protocol 2). ﻿To evaluate the functional significance, proteins identified by ≥ 2 unique validated peptides were used. The most enriched relevant biological processes (*P* < 0.01; at alpha level 0.05) in HaCaT cells treated with both sample preparation Protocol 1 and Protocol 2 were “Nuclear mRNA splicing, via spliceosome”, “Epithelial cell differentiation” (Fig. 4a). GO analysis suggested that “slicing, via spliceosome” was slightly activated due to SDS exposure. Earlier, it was shown that alternative splicing induction may contribute to the changing proteomic landscape as at carcinogenesis, for example, in HaCaT cells after arsenic exposure^33^. In contrast, several metabolic pathways, such as negative regulation of apoptotic process and epidermis development, were downregulated after treatment of HaCaT cells with SDS. Nevertheless, by conventional criteria, all the differences were considered to be not statistically significant, apparently, because doses of SDS were non-toxic (25 μg/mL) and did not lead to cell death during 48 h of exposure^6^.

The most enriched cell components were “cytosol”, “nucleus", and “mitochondrion” (Fig. 4b). As a main result, the Protocol 1 proteome demonstrated to contain a higher percentage of membrane proteins, while the Protocol 2 proteome was enriched in cytosol proteins. The higher number of membrane proteins was in agreement with SDS solubilizing activity^34^. Overall, the proportions of cellular components were preserved during the treatment of HaCaT keratinocytes with SDS.

### Human keratinocyte-specific proteins

In the context of our comparison of different sample preparation protocols, it should be emphasized that the problem of protein identification number is an inherent part of the bottom-up strategy. The success of sample preparation may be assessed through the number of revealed tissue-specific proteins. However, it would be more reliable to compare tissue-specific proteins and/or whole protein families detected with different approaches. For the HaCaT cells, keratinocyte-specific proteins may be used for comparison.

Herein, we identified 60 human keratinocyte-specific proteins (Supplementary Table S5), which is equal to about 11% of all proteins denoted to basal and suprabasal keratinocytes, according to the Human Protein Atlas. Among them, 55 proteins were detected utilizing the 0.2% SDS lysis with 1DE-gel concentration (Protocol 1). The gel-free approach (Protocol 2) revealed only 38 keratinocyte-specific proteins. A smaller number of detected specific proteins in the case of Protocol 2 was, for certain reasons, partly due to the osmotic lysis resolution.

At each stage of differentiation, keratinocytes express specific keratins and other markers, such as involucrin, loricrin, transglutaminase, filaggrin, and caspase 14^1,35^. For example, utilizing sample preparation Protocol 1, we have exclusively detected filaggrin (FLG) and caspase-14 (CASP14) in control HaCaT cells (Supplementary Table S5). The detection of FLG might be linked with reaching cell confluence since, during cultivation, the differentiation process may be induced. So FLG may be detected in low concentrations in undifferentiated cells, including the HaCaT line^36,37^. In agreement, we detected FLG with low NSAF value only in one out of the three technical repeats. According to our data, FLG and CASP-14 were captured in the first 25% quartile of the proteins with NSAF values very close to the bottom dynamic range for NSAF value counting^31^. Therefore, we had assumed that sample processing based on the SDS extraction in conjunction with a consequent 1DE-gel concentration approach exhibited sufficient sensitivity essential for the studies of human cell culture proteins.

Usually, in the analysis of cellular responses, the results of assessing gene expression are normalized to a reference gene^38^. The endogenous control is typically a housekeeping gene, which, ideally, is uniformly expressed during all environmental or experimental conditions in the given experimental system. The range of expression should be similar to the target gene analyzed. The most commonly used reference genes in keratinocytes are glyceraldehyde-3-phosphate dehydrogenase (*GAPDH*), hypoxanthine-guanine-phosphoribosyl-transferase (*HPRT*), phosphoglycerate kinase 1 (*PGK1*), and tubulin (*TBB*)^39,40^. Obviously, these rules can also be applied to assess protein abundance under different experimental conditions. It turned out that the range of NSAF values of keratinocyte-specific proteins detected in HaCaT cells was comparable to NSAF values of keratinocyte housekeeping proteins (Table 1) that underwent the same preparation steps.

**Table 1:** Housekeeping proteins identified in HaCaT keratinocytes. Protocol 1—0.2% SDS lysis with 1DE-gel concentration approach; Protocol 2—osmotic shock (H_2_O-based) extraction.

##	Accession	Gene	Description	Function	NSAF value			
					Protocol 1		Protocol 2	
					Control	SDS-exposed	Control	SDS-exposed
1	P04406	*GAPDH*	Glyceraldehyde-3-phosphate dehydrogenase	Glycolysis	0.528	0.536	1.030	2.083
2	P00492	*HPRT*	Hypoxanthine-guanine-phosphoribosyl-transferase	Generation of purine nucleotides	0.042	0.058	0.026	0.056
3	P00558	*PGK1*	Phosphoglycerate kinase 1	Glycolysis	0.142	0.205	0.284	0.497
4	P07437	*TBB5*	Tubulin beta chain	GTP-binding	0.156	0.223	0.699	1.328

In such a way, the obtained results attested that two sample preparation approaches compared in this study are eligible for assessing the protein abundance changes introduced by HaCaT SDS treatment.

### Comparative proteoinformatics pipeline demonstrated the utility of Protocol 1

HaCaT cell line may be a useful model for investigating anti-inflammatory interventions/therapies for skin diseases^41–43^. Moreover, HaCaT keratinocytes are used for the studies of multistep carcinogenesis in human cells^44^. Herein, we have tested the earlier proposed hypothesis^6^ that relatively prolonged (48 h) non-toxic (25 μg/mL) SDS skin exposure might be associated with the initiation of malignancies, using HaCaT cells.

**Figure 5:**
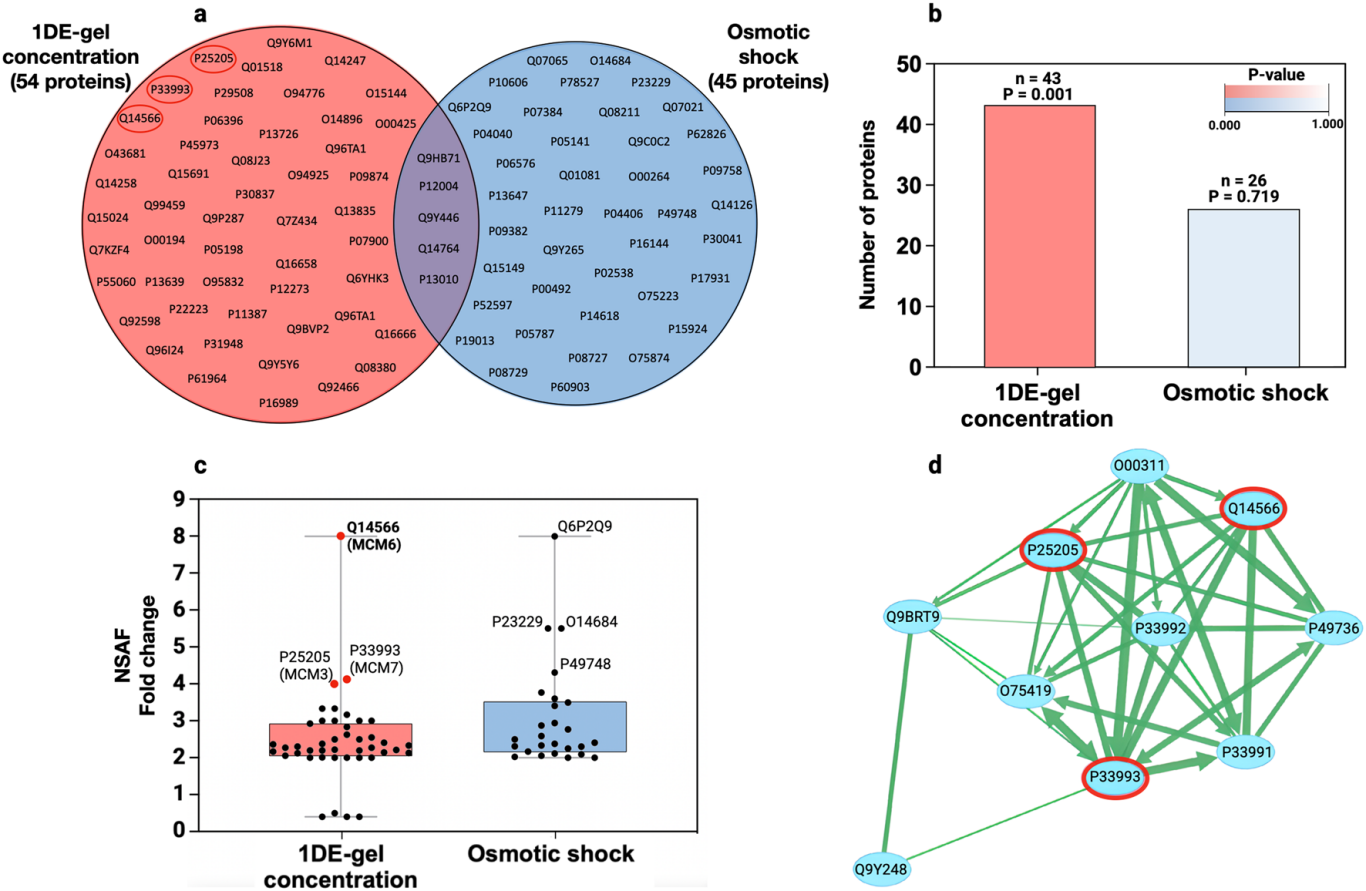
Comparative proteoinformatics pipeline. (**a**) Venn diagram illustrating the distribution of up- and downregulated (≥ 2 times) proteins associated with cellular proliferation disease (DOID:14566); (**b**) the number of skin cancer associated proteins (according to COSMIC database: https://cancer.sanger.ac.uk/cosmic, accessed on 21 June 2022); (**c**) NSAF fold change distribution of skin cancer associated proteins (COSMIC database); (**d**) Protein–protein interaction (PPI) network of double-strand break repair via break-induced replication pathway (GO:0,000,727). The skin cancer associated proteins (COSMIC database) identified with Protocol 1 (0.2% SDS solubilization + 1DE-gel concentration) are circled in red.

To reveal the proteins and/or pathways related to the SDS impact on human skin keratinocytes, we have suggested a comparative bioinformatics pipeline consisting of several consecutive stages. First, we created two lists of common proteins for control and SDS-treated cells, both for Protocol 1 (n = 778) and Protocol 2 (n = 254). Then, the proteins with a fold change ≥ 2 times (up- and downregulated) in the value of NSAF were selected and mapped to corresponding genes using the UniProt database (v. 23.06.2022, https://www.uniprot.org/uploadlists/). The resulting gene lists were analyzed using the Disease Ontology database (DO)^45^. As a result, it turned out that for Protocol 1, a total of 82 genes/proteins were involved in pathological processes that exist in an organism (Disease, DOID:4), of which 54 were connected to the disease of cellular proliferation (DOID:14566). Protocol 2 allowed us to find 60 genes/proteins of disease ontology (DOID:4), of which 45 were involved in the disease of cellular proliferation (DOID:14566). Only a little overlap (five common genes/proteins) was apparent among the resulting lists (Fig. 5a). In the next step, the protein lists associated with the disease of cellular proliferation were analyzed against the COSMIC (Catalogue of Somatic Mutations in Cancer) database^46^. As a result, skin cancer was the most substantial COSMIC term. Comparative characteristics of Protocols 1 and 2 in terms of the number of proteins associated with skin cancer and their distributions depending on NSAF fold change are presented in Fig. 5b, c. At the final stage, the analysis of the found skin cancer-related protein lists consisting of 43 and 27 proteins for Protocols 1 and 2, respectively, was carried out using an open-source software platform Cytoscape (assessed on 27.06.2022, https://cytoscape.org). Those proteins identified with Protocol 1 were involved (*P * = 3.84e−8; at alpha level 0.05) in double-strand break repair (BIR) via break-induced replication pathway (GO:0000727), whereas Protocol 2 did not reveal any significant pathways on 27 MS identifications associated with skin cancer.

As can be seen from Fig. 5c,d, in Protocol 1 (43 proteins), we could detect three upregulated members of the MCM family associated with BIR in HaCaT cells after SDS treatment—MCM3 (P25205), MCM6 (Q14566), and MCM7 (P33993). Minichromosome maintenance (MCM) proteins are significant DNA replication regulators that occur in a precise fashion during eukaryotic cell division. Highly conserved hexameric complexes of DNA-binding proteins of the MCM family have six subtypes, namely, MCM2, MCM3, MCM4, MCM5, MCM6 and MCM7. The MCM proteins are essential for proliferating cells. The dysregulation of these proteins directly influences the DNA replication system and is thus involved in the initiation, development and progression of cancer, autoimmune diseases and other diseases^47^. Additionally, according to the literature, MCMs are frequently upregulated in a variety of dysplastic and cancer cells^48–50^.

Among the upregulated MCMs detected exclusively with Protocol 1 (Fig. 5c, red dots), MCM6 showed the highest increase in NSAF value as a response to SDS exposure. The NSAF value for MCM6 was increased eightfold, while for MCM3 and MCM7, NSAF values were enhanced four times for each family member. A manual review of the data presented in the protein-centric knowledge platform neXtProt (release 2022-05-19, https://www.nextprot.org) for MCM3, MCM6, and MCM7 expression in the keratinocytes shows their expression at the mRNA level but not at the protein level. We managed to show the existence of these proteins in HaCaT keratinocytes and their alterations upon SDS exposure by sample processing approach based on 0.2% SDS-solubilization and 1DE-gel clean-up procedure (sample preparation Protocol 1) prior to LC-MS/MS.

The existence of MCM6 has been shown by nine validated unique peptides (Table 2), seven of which belong to the category “natural + synthetic”. That is, they are both “natural” (have been detected in biological samples) and chemically synthesized as reagents for selected reaction monitoring (SRM) experiments (“synthetic”). For example, the peptide ^270^*VSGVDGYETEGIR*^282^ was used in SRM assays to validate the MCM6 upregulated expression induced by 17β-estradiol in MCF-7 breast cancer cells^51^. The ^589^*ESEDFIVEQYK*^599^ was used in proteome-based platforms for identifying potential prognostic biomarkers for the stratification of ER-positive breast cancer patients into groups of low and high risk for disease recurrence^52^.

**Table 2:** The list of identified by SearchGUI peptides (sample preparation Protocol 1), which belong to DNA replication licensing factor MCM6 (MCM6_HUMAN, Q14566). NaN—m/z error in ppm equal 0.

##	Sequence	Position	Length	Category	Control HaCaT cells			SDS-exposed HaCaT cells		
					m/z exp	Charge	m/z error in ppm	m/z exp	Charge	m/z error in ppm
1	*YLQLAEELIRPER*	46–58	13	Natural + synthetic	nd	–	–	543.97	3 +	NaN
2	*DFYVAFQDLPTR*	109–120	12	Natural + synthetic	736.37	2 +	1.24	736.87	2 +	NaN
3	*IQETQAELPR*	208–217	10	Natural + synthetic	nd	–	–	592.82	2 +	NaN
4	*VSGVDGYETEGIR*	270–282	13	Natural + synthetic	nd	–	–	691.33	2 +	NaN
5	*GDINVCIVGDPSTAK*	388–402	15	Natural	nd	–	–	773.38	2 +	NaN
6	*TSILAAANPISGHYDR*	497–512	16	Natural + synthetic	nd	–	–	562.63	3 +	NaN
7	*ESEDFIVEQYK*	589–599	11	Natural + synthetic	nd	–	–	693.83	2 +	NaN
8	*ISNLIVLHLR*	723–732	10	Natural	793.25	3 +	1.23	793.25	3 +	NaN
9	*SELVNWYLK*	746–754	9	Natural + synthetic	nd	–	–	576.31	2 +	NaN

It has been recently assumed that MCM6 may represent a protein that can be linked with the development of multiple cancer types^53^. Moreover, MCM6 overexpression may predict the poor survival of patients with one of many cancer types, such as glioma^54^, hepatocellular carcinoma^55^ and endometrioid endometrial adenocarcinoma^56^. As a member of the double-strand break repair pathway (GO:0000727), MCM6 protein exhibited considerable upregulation in SDS-exposed HaCaT keratinocytes upon our experimental design. It is known from the literature that DNA damage leads to genetic instability, which in turn may enhance the rate of cancer development. The DNA-damage repair pathways are fundamental to the etiology of most, if not all, human cancers^57^. Finally, we speculate that MCM6 protein may be the "checkpoint" of the possible SDS-initiated proteomic landscape changes in human skin including those leading to the occurrence of cutaneous tumorigenesis.

### Relative mRNA expression analysis of *MCM6* by reverse transcription quantitative PCR

**Figure 6:**
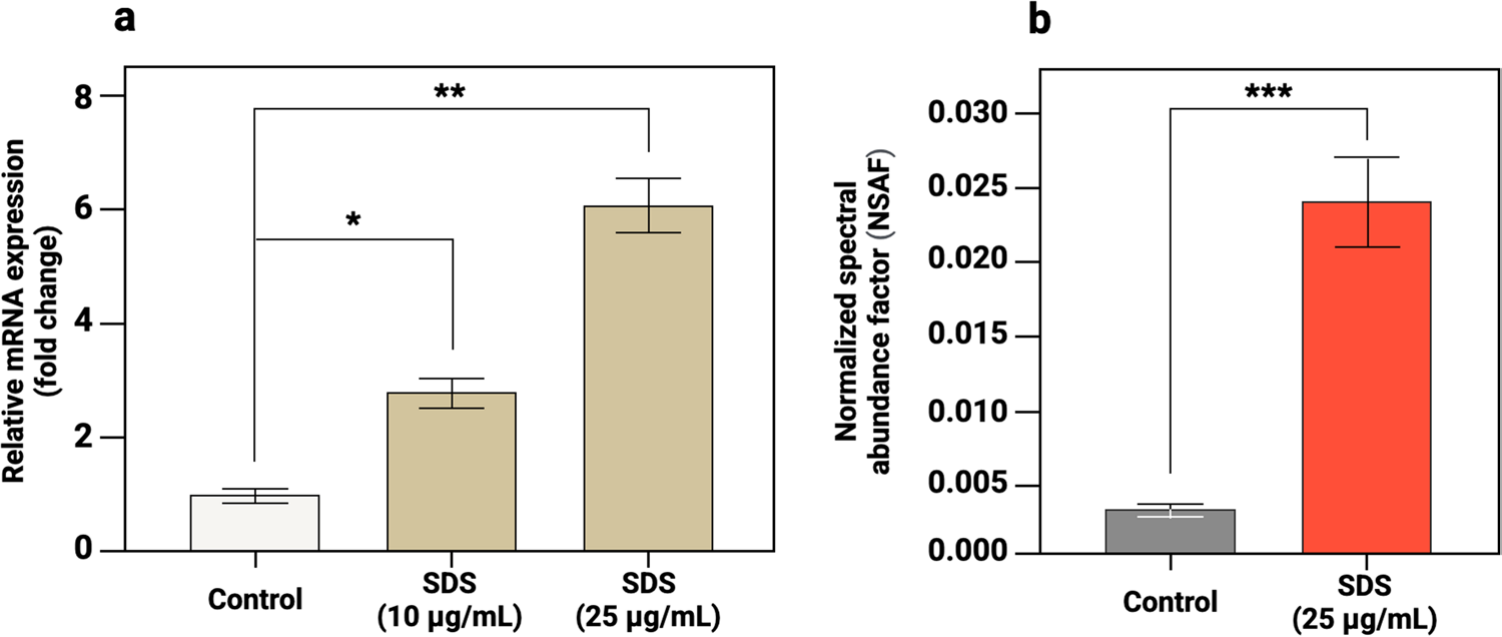
The mRNA expression levels of *MCM6* and corresponding MCM6 proteomic profiles in control and SDS-exposed HaCaT cells. Histograms show (**a**) the fold change in *MCM6* expression in SDS-exposed cells compared to control cells; (**b**) protein abundance of MCM6 assessed via LC–MS/MS analysis. The significant upregulations are marked with asterisks (*P = 3.8e−5; **P = 2.8e−5; ***P = 3.0e−4; at alpha level 0.05). Error bars represent the standard deviation between technical repeats.

In order to verify the upregulation of MCM6 registered via LC-MS/MS assays, we decided to use mRNA level as an independent proxy^58^ for where to expect high versus low abundance of MCM6. To assess the relative mRNA expression of *MCM6*, reverse transcription quantitative PCR (RT-qPCR) analysis was performed (Fig. 6).

Treatment with SDS resulted in a significant increase (*P* < 0.001, at alpha level 0.05) in *MCM6* expression at mRNA level in a dose-dependent manner. The fold change was 2.8 and 6.1 for 10 µg/mL and 25 µg/mL SDS, respectively (Fig. 6a). Summarizing, the transcription levels of *MCM6* gene were consistent with our mass-spectrometric assays by which we have obtained an eightfold increase in MCM6 content (Fig. 6b), in response to non-toxic concentration (25 μg/mL) during relatively prolonged (48 h) SDS exposure. Our results corresponded to literature data reporting cases of differential expression when mRNAs were sufficiently correlated with respective protein products^59^. At least two examples show that the expression level of *MCM6* has been increased conjunctively with proteome level in neuroblastoma^60^ and clear-cell renal cell carcinoma^61^.

The obtained results reinforce the previous observations about the possible effects of SDS exposure on the development of adverse effects in human skin keratinocytes. Previously, we have shown that keratinocyte SDS exposure (25 μg/mL) leads to a decrease in advanced glycation end-products^62^, which are the generally accepted oxidative stress markers correlating with the progression of various types of cancer^63^. Thus, the reported findings of Protocol 1 are viable for understanding the potential initiation of skin disease pathways due to the use of SDS-containing household chemicals, cosmetic products, shampoos, solvents, and conditioning skin agents.

In conclusion, the two proteomic sample preparation protocols did not show clear differences when compared by the routinely used protein identification parameters (sequence coverage, number of validated unique peptides, NSAF values characterizing proteins). Whilst Protocol 2 (cell lysis by osmotic shock) caused lower protein degradation Protocol 1 (0.2% SDS-based solubilization combined with 1DE-gel concentration) stood out with the versatile profiling (enhanced number of total identifications, low-abundance proteins, and proteins previously not detected at the protein level in keratinocytes). Generally, the proteome characterization by GO analysis did not reveal significant differences; all peculiarities lay within the specificity of protocol frameworks. Nonetheless, while assessing from the «eagle eye» viewpoint, our work demonstrated the power of Protocol 1 in the characterization of HaCaT cell proteome and identifying non-obvious features of SDS impact. Notwithstanding the utilized SDS dose does not lead to a decrease in HaCaT cell viability, we revealed the upregulation of a double-strand break repair via break-induced replication pathway (GO:0000727). The pathway was detected at a significant level (*P * = 3.84e−8; at alpha level 0.05) by three proteins of the minichromosome maintenance (MCM) family (MCM3, MCM6, and MCM7) that show a potential to discriminate between HaCaT cells with and without SDS treatment. The role of MCM6 overexpression in the development of adverse effects (e.g., the development of neoplastic processes) due to SDS requires further investigation, i.e., verification and validation^64^. Practically, verification is focused on confirming the abundances of target proteins and/or peptides significantly distinct in disease compared to control groups with quantitative measurements (for example, immunoblotting, single reaction monitoring mass spectrometry, etc.). The validation is directed to reinforce the verification results by a large cohort of HaCaT cells and different cancer cell lines (for example, skin cancer cell lines A-431, SK-MEL, WM-115, lymphoblastoid cell line MOLT-4). Such a pool of studies could permit the assessment of the possible carcinogenic effect of SDS on the skin. Herein, the comparative proteoinformatics pipeline led us to novel insights into the oncogenic potential of immortalized HaCaT cell line that has been under relatively prolonged SDS treatment. This view expands the fundamentals of using HaCaT cells in various applications, including the detection of important aspects of cancer development.

## Methods

### Cell cultivation and SDS exposure

Immortalized HaCaT keratinocytes (from Bank of DKFZ, Heidelberg) before (control) and after exposure to SDS (25 μg/mL) during 48 h were used in the study. Cell cultivation and SDS exposure were carried out as described earlier^6^. Detailed description is given in Supplementary file “Supporting Information” in “Cell cultivation and SDS exposure” section.

### Protein extraction

In order to ensure sufficient HaCaT protein concentration for proteomic analysis in this study, we pooled the cells from three flasks (three biological replicates for each group: control and SDS-exposed, treated with Protocols 1 and 2) in a single tube for further processing.

Protocol 1—HaCaT cell pellet was placed into 500 μL of 0.2% SDS in 100 mM Tris-HCl (pH = 7.4), 120 mM NaCl, 5 mM EDTA, 1% PMSF and manually homogenized in a glass homogenizer. After sonication (in an ice-cold bath, active time 25 s), the samples were incubated for 30 min at + 4 °C on an orbital shaker with a platform rotation of 1000 rpm. After heating at 95 °C for 4 min, it was centrifuged at 14,000 × g for 20 min (+ 4 °C). The lysate was collected, and the procedure was repeated starting from the sonication step. Lysates were pooled and centrifuged at 14,000 × g for 60 min (+ 4 °C); the finished supernatant was collected.

Protocol 2—The water cell homogenate of HaCaT keratinocytes (180 μL of cold water, 65 mM DDT, and 1% protease inhibitor E64, freshly prepared every time) was prepared by sonication as in Protocol 1. The lysate was centrifuged at 15,000 × g at + 4 °C for 15 min twice to remove debris.

The total protein concentration of HaCaT extracts was determined by the bicinchoninic acid assay^65^ on an Agilent 8453 UV–visible spectrophotometer with BSA as a standard.

### 1DE-gel concentration and in-gel digestion

The 1DE-gel concentration protocol was then carried out to remove SDS as described earlier^21^. In-gel digestion with trypsin was performed according to the standard procedure^66^. Detailed description is given in Supplementary file “Supporting Information” in “1DE-gel concentration and in-gel digestion” section.

The mixture of proteolytic peptides from each gel band was used for liquid chromatography coupled with tandem mass spectrometry (LC–MS/MS) analysis.

### In-solution tryptic digestion

The pair of water extracts (175 μg of protein) for each study group, namely control and SDS-exposed HaCaT cells, were in-solution digested in accordance with the protocol described earlier^21^. Additional details are provided in Supplementary file “Supporting Information” in “In-solution tryptic digestion” section.

Peptide digest mixtures were analyzed without further processing using LC–MS/MS.

### LC–MS/MS analysis

Separation and identification of the peptides were performed on an Ultimate 3000 nano-flow HPLC system (Dionex, USA), connected to a Orbitrap Q Exactive mass-spectrometer (Thermo Scientific, USA) equipped with a Nanospray Flex NG ion source (Thermo Scientific, USA)^21^. Detailed information is given in Supplementary file “Supporting Information” in “LC–MS/MS analysis” section.

### Reverse transcription quantitative PCR

HaCaT cells were cultured under standard conditions, harvested by trypsinization and seeded at the density of 2.0 × 104 cells per 60 mm Petri dishes. After 24 h, the cultured cells were divided into three groups: one was control (fresh medium was added), and two others were exposed to SDS (10 µg/mL or 25 µg/mL), and then followed by 48 h of incubation under culture conditions. For every sample group, there were two independent biological replicates, each performed in triplicate. Total RNA was isolated with the RNeasy kit (Qiagen, Netherlands) following the manufacturer’s instructions. cDNA synthesis was performed via MMLV reverse-transcription kit (Evrogen, Russia) utilizing 1 µg of total RNA per reaction. Reverse transcription quantitative PCR (RT-qPCR) analysis was performed with SYBR label (Evrogen) in Bio-rad CFX connect system. Relative expression of *MCM6* was calculated with the ΔΔCt method^67^ using *GAPDH* and *ACTB* as reference genes^68^. The primers employed for analysis are listed in Table 3. Raw data were analyzed using CFX Maestro 1.0 software (v. 4.0.0325.0418). One-way analysis of variance was performed using the Tukey HSD test for the multiple comparisons of mean values^69^.

**Table 3:** Primer sequences for the studied genes.

Target	Forward primer	Reverse primer
*GAPDH*	5′-TCG ACA GTC AGC CGC ATC TTC TTT-3′	5′-ACC AAA TCC GTT GAC TCC GAC CTT-3′
*ACTB*	5′-TCA GAA GGA TTC CTA TGT GGG CGA-3′	5′-CAC GCA GCT CAT TGT AGA AGG TGT-3′
*MCM6*	5′-GTG ATC AGG GAT GTA GAA CAG C-3′	5′-AGC TTG GGT CTC TTG AAT ACG-3′

### Data processing

The initial RAW files were converted to MGF files with the ProteoWizard MSConvert program (v. 3.0.20310, https://proteowizard.sourceforge.io). Files were imported into the SearchGUI (v. 4.1.8) platform^70^, the processing was performed with X!Tandem and MS-GF + search algorithms against the UniProt human database (v. 22.03.2022, https://www.uniprot.org/proteomes/UP000005640, FASTA format). The following search parameters were set: enzyme — trypsin; the maximum number of missed cleavages—1; fixed modification—piridylethylation of C (in-solution tryptic digestion) or carbamidomethylation of C (in-gel digestion); variable modification—oxidation methionine; parent and fragment ions tolerances— ± 5 ppm and ± 0.01 Da, respectively. The PeptideShaker integrator^71^ was utilized to generate Excel spreadsheet tables with the results that contain values of sequence coverage (%), the number of validated unique peptides for protein, normalized spectral abundance factor (NSAF) and so on. In this study, NSAF values were chosen for quantifying the proteins, due to the demonstrated capability of NSAF method being highly reproducible^72^. So then NSAF values also provide the ability to compare the abundance of proteins within a sample and/or between samples^73^.

A list of typical protein contaminants was obtained from the common Repository of Adventitious Proteins^29^.

Results were imported into Funrich software^74^ to build Venn diagrams and perform GO enrichment analysis (database v. 12.12.2021, http://geneontology.org/docs/downloads/) and COSMIC search (against database v. 21.06.2022, https://cancer.sanger.ac.uk/cosmic). The R language (v. 2022.02.0 + 443, https://www.r-project.org) packages were applied to visualize violin plots (beanplot, v. 1.3.1^75^) and process Disease Ontology analysis—(DOSE, v. 3.20.1^76^). NSAF fold change distribution presented in box-and-whiskers plot was built with Prism 9 GraphPad (v. 9.4.0, https://www.graphpad.com/scientific-software/prism/). Cytoscape platform (https://cytoscape.org, accessed on 05.07.2022) was used to build protein–protein interaction network.

## Supplementary Information

Supplementary Information 1.

Supplementary Information 2.

Supplementary Information 3.

Supplementary Information 4.

Supplementary Information 5.

Supplementary Information 6.

## Data Availability

The datasets generated and/or analysed during the current study are available in the ProteomeXchange repository, http://proteomecentral.proteomexchange.org/cgi/GetDataset?ID=PXD035202.

## References

[1] Breitkreutz, D. *et al.* Human keratinocyte cell lines, in *Pharmaceutical Applications of Cell and Tissue Culture to Drug Transport* (eds Wilson, G., Davis, S.S., Illum, L., Zweibaum, A.) 283–296 (NATO ASI Series, Springer, 1991).

[2] OECD Test no. 439: In vitro skin irritation: reconstructed human epidermis test method, in *OECD Guidelines for the Testing of Chemicals* Section 4 (OECD Publishing, 2021).

[3] Rusanov AL, Luzgina NG, Lisitsa AV (2017). Sodium dodecyl sulfate cytotoxicity towards HaCaT keratinocytes: Comparative analysis of methods for evaluation of cell viability. Bull. Exp. Biol. Med..

[4] Singer MM, Tjeerdema RS (1993). Fate and effects of the surfactant sodium dodecyl sulfate. Rev. Environ. Contam. Toxicol..

[5] Bondi CA (2015). Human and environmental toxicity of sodium lauryl sulfate (SLS): Evidence for safe use in household cleaning products. Environ. Health Insights.

[6] Petushkova N (2020). Proteomic characterization of HaCaT keratinocytes provides new insights into changes associated with SDS exposure. BMC Dermatol..

[7] Simonnet H (2002). Low mitochondrial respiratory chain content correlates with tumor aggressiveness in renal cell carcinoma. Carcinogenesis.

[8] Elguoshy A (2016). Why are they missing?: Bioinformatics characterization of missing human proteins. J. Proteom..

[9] Grabski AC (2009). Advances in preparation of biological extracts for protein purification. Methods Enzymol..

[10] Shehadul Islam M, Aryasomayajula A, Selvaganapathy PR (2017). A review on macroscale and microscale cell lysis methods. Micromachines.

[11] Malherbe G, Humphreys DP, Davé E (2019). A robust fractionation method for protein subcellular localization studies in Escherichia coli. Biotechniques.

[12] Brown RB, Audet J (2008). Current techniques for single-cell lysis. J. R. Soc. Interface.

[13] Peach M, Marsh N, Miskiewicz EI, MacPhee DJ (2015). Solubilization of proteins: The importance of lysis buffer choice. Methods Mol. Biol..

[14] Assay Genie Sonication Protocol for Cell Lysis. *Assay Genie Website* https://www.assaygenie.com/sonication-protocol-for-cell-lysis/ (2022).

[15] Kisrieva YS (2020). Comparative study of the human keratinocytes proteome of the HaCaT line: Identification of proteins encoded by genes of 18 chromosomes under the influence of detergents. Biomed. Khim..

[16] Hughes CS (2019). Single-pot, solid-phase-enhanced sample preparation for proteomics experiments. Nat. Protoc..

[17] Litovchick L (2018). Preparing whole-cell lysates for immunoblotting. Cold Spring Harb. Protoc..

[18] Quirino JP (2018). Sodium dodecyl sulfate removal during electrospray ionization using cyclodextrins as simple sample solution additive for improved mass spectrometric detection of peptides. Anal. Chim. Acta.

[19] Yeung YG, Nieves E, Angeletti RH, Stanley ER (2008). Removal of detergents from protein digests for mass spectrometry analysis. Anal. Biochem..

[20] Kurien BT, Aggarwal R, Scofield RH (1855). Protein extraction from gels: A brief review. Methods Mol. Biol..

[21] Shkrigunov T (2022). Protocol for increasing the sensitivity of MS-based protein detection in human chorionic villi. Curr. Issues Mol. Biol..

[22] Doellinger J, Schneider A, Hoeller M, Lasch P (2020). Sample preparation by easy extraction and digestion (SPEED)—A universal, rapid, and detergent-free protocol for proteomics based on acid extraction. Mol. Cell. Proteomics.

[23] Merck KGaA Solubilization of membrane proteins. *Merck KGaA Website* https://www.sigmaaldrich.com/RU/en/technical-documents/protocol/protein-biology/protein-purification/solubilization (2022).

[24] Goldman AR (2019). Proteome analysis using gel-LC-MS/MS. Curr. Protoc. Protein Sci..

[25] Xiong Y, Zhang Y, Yao J, Yan G, Lu H (2018). A streamlined sample preparation method for mass spectrometric analysis. Curr. Protoc. Cell Biol..

[26] Muinao T, Pal M, Boruah H (2018). Cytosolic and transmembrane protein extraction methods of breast and ovarian cancer cells: A comparative study. J. Biomol. Tech..

[27] Middelberg AP (1995). Process-scale disruption of microorganisms. Biotechnol. Adv..

[28] Mæhre HK, Jensen IJ, Eilertsen KE (2016). Enzymatic pre-treatment increases the protein bioaccessibility and extractability in dulse (*Palmaria palmata*). Mar. Drugs.

[29] The Global Proteome Machine Organization Common Repository of Adventitious Proteins. *The Global Proteome Machine website* https://www.thegpm.org/crap/ (2012).

[30] Hintze JL, Nelson RD (1998). Violin plots: A box plot-density trace synergism. Am. Stat..

[31] Neilson KA, Keighley T, Pascovici D, Cooke B, Haynes PA (2013). Label-free quantitative shotgun proteomics using normalized spectral abundance factors. Methods Mol. Biol..

[32] de Winter JCF (2013). Using the Student’s t-test with extremely small sample sizes. Pract. Assess. Res. Eval..

[33] Ferragut Cardoso AP (2022). Temporal modulation of differential alternative splicing in HaCaT human keratinocyte cell line chronically exposed to arsenic for up to 28 wk. Environ. Health Perspect..

[34] Lin Y (2013). Evaluation of the combinative application of SDS and sodium deoxycholate to the LC-MS-based shotgun analysis of membrane proteomes. J. Sep. Sci..

[35] Chieosilapatham P (2021). Keratinocytes: Innate immune cells in atopic dermatitis. Clin. Exp. Immunol..

[36] Resing KA, Al-Alawi N, Blomquis C, Fleckman P, Dale B (1993). Independent regulation of two cytoplasmic processing stages of the intermediate filament-associated protein filaggrin and role of Ca2+ in the second stage. J. Biol. Chem..

[37] Miyoshi S, Yamazaki S, Uchiumi A, Katagata Y (2012). The Hsp90 inhibitor 17-AAG represses calcium-induced cytokeratin 1 and 10 expression in HaCaT keratinocytes. FEBS Open Bio.

[38] Bermudez Y (2011). Nicotinic acid receptor abnormalities in human skin cancer: Implications for a role in epidermal differentiation. PLoS ONE.

[39] Cabiati M (2012). Tissue-specific selection of stable reference genes for real-time PCR normalization in an obese rat model. J. Mol. Endocrinol..

[40] Hu X (2016). Common housekeeping proteins are upregulated in colorectal adenocarcinoma and hepatocellular carcinoma, making the total protein a better “housekeeper”. Oncotarget.

[41] Fusenig NE, Boukamp P (1998). Multiple stages and genetic alterations in immortalization, malignant transformation, and tumor progression of human skin keratinocytes. Mol. Carcinog..

[42] Li K (2019). Anthocyanins from black peanut skin protect against UV-B induced keratinocyte cell and skin oxidative damage through activating Nrf 2 signaling. Food Funct..

[43] Colombo I (2017). HaCaT cells as a reliable in vitro differentiation model to dissect the inflammatory/repair response of human keratinocytes. Mediat. Inflamm..

[44] Boukamp P (1988). Normal keratinization in a spontaneously immortalized aneuploid human keratinocyte cell line. J. Cell Biol..

[45] Schriml LM (2022). The human disease ontology 2022 update. Nucl. Acids Res..

[46] Tate JG (2019). COSMIC: The catalogue of somatic mutations in cancer. Nucl. Acids Res..

[47] Freeman A (1999). Minichromosome maintenance proteins as biological markers of dysplasia and malignancy. Clin. Cancer Res..

[48] Harada H (2008). Cleavage of MCM2 licensing protein fosters senescence in human keratinocytes. Cell Cycle.

[49] Lameira AG (2014). MCM3 could be a better marker than Ki-67 for evaluation of dysplastic oral lesions: An immunohistochemical study. J. Oral Pathol. Med..

[50] Choy B, LaLonde A, Que J, Wu T, Zhou Z (2016). MCM4 and MCM7, potential novel proliferation markers, significantly correlated with Ki-67, Bmi1, and cyclin E expression in esophageal adenocarcinoma, squamous cell carcinoma, and precancerous lesions. Hum. Pathol..

[51] Drabovich AP, Pavlou MP, Schiza C, Diamandis EP (2016). Dynamics of protein expression reveals primary targets and secondary messengers of estrogen receptor alpha signaling in MCF-7 breast cancer cells. Mol. Cell. Proteomics.

[52] Pavlou MP (2014). Integrating meta-analysis of microarray data and targeted proteomics for biomarker identification: Application in breast cancer. J. Proteome Res..

[53] Zeng T (2021). The DNA replication regulator MCM6: an emerging cancer biomarker and target. Clin. Chim. Acta.

[54] Cai HQ (2018). Overexpression of MCM6 predicts poor survival in patients with glioma. Hum. Pathol..

[55] Liu Z (2018). MCM family in HCC: MCM6 indicates adverse tumor features and poor outcomes and promotes S/G2 cell cycle progression. BMC Cancer.

[56] Hotton J (2018). Minichromosome maintenance complex component 6 (MCM6) expression correlates with histological grade and survival in endometrioid endometrial adenocarcinoma. Virchows Arch..

[57] Khanna KK, Jackson SP (2001). DNA double-strand breaks: Signaling, repair and the cancer connection. Nat. Genet..

[58] Sivertsson Å (2020). Enhanced validation of antibodies enables the discovery of missing proteins. J. Proteome Res..

[59] Koussounadis A, Langdon SP, Um IH, Harrison DJ, Smith VA (2015). Relationship between differentially expressed mRNA and mRNA-protein correlations in a xenograft model system. Sci. Rep..

[60] Gu Y (2021). MCM6 indicates adverse tumor features and poor outcomes and promotes G1/S cell cycle progression in neuroblastoma. BMC Cancer.

[61] Jang NR, Baek J, Ko Y, Song PH, Gu MJ (2021). High MCM6 expression as a potential prognostic marker in clear-cell renal cell carcinoma. In Vivo.

[62] Petushkova N (2017). Proteome of the human HaCaT keratinocytes: Identification of the oxidative stress proteins after sodium dodecyl sulpfate exposur. Mol. Cell Biol..

[63] Kuniyasu H (2002). Expression of receptors for advanced glycation end-products (RAGE) is closely associated with the invasive and metastatic activity of gastric cancer. J. Pathol..

[64] Nakayasu ES (2021). Tutorial: best practices and considerations for mass-spectrometry-based protein biomarker discovery and validation. Nat. Protoc..

[65] Smith P (1985). Measurement of protein using bicinchoninic acid. Anal. Biochem..

[66] Shevchenko A, Wilm M, Vorm O, Mann M (1996). Mass spectrometric sequencing of proteins silver-stained polyacrylamide gels. Anal. Chem..

[67] Livak KJ, Schmittgen TD (2001). Analysis of relative gene expression data using real-time quantitative PCR and the 2(-Delta Delta C(T)) method. Methods.

[68] Sun Y, Li Y, Luo D, Liao DJ (2012). Pseudogenes as weaknesses of ACTB (Actb) and GAPDH (Gapdh) used as reference genes in reverse transcription and polymerase chain reactions. PLoS ONE.

[69] Tukey JW, Ciminera JL, Heyse JF (1985). Testing the statistical certainty of a response to increasing doses of a drug. Biometrics.

[70] Vaudel M, Barsnes H, Berven F, Sickmann A, Martens L (2011). SearchGUI: an open-source graphical user interface for simultaneous OMSSA and X!Tandem searches. Proteomics.

[71] Vaudel M (2015). PeptideShaker enables reanalysis of MS-derived proteomics data sets. Nat. Biotechnol..

[72] McIlwain S (2012). Estimating relative abundances of proteins from shotgun proteomics data. BMC Bioinform..

[73] Oldach M. Normalized spectral abundance factor (NSAF) for quantitative liquid chromatography mass spectrometry-based proteomics. *GitHub* https://github.com/moldach/proteomics-spectralCount-normalization (2018).

[74] Fonseka P, Pathan M, Chitti SV, Kang T, Mathivanan S (2021). FunRich enables enrichment analysis of OMICs datasets. J. Mol. Biol..

[75] Kampstra, P. Beanplot: Visualization via beanplots (like boxplot/stripchart/violin plot). *The Comprehensive R Archive Network* https://CRAN.R-project.org/package=beanplot (2022).

[76] Yu G, Wang LG, Yan GR, He QY (2015). DOSE: an R/Bioconductor package for disease ontology semantic and enrichment analysis. Bioinformatics.

