# Supplementary material for: Comparative proteoinformatics revealed the essentials of SDS impact on HaCaT keratinocytes
Source: Sci Rep. 2022 Dec 12;12:21437. doi: 10.1038/s41598-022-25934-4 (PMC9744838; doi:10.1038/s41598-022-25934-4)
Supplement: Supplementary file 1 — Supplementary Information 1. [file 41598_2022_25934_MOESM1_ESM.pdf]

Comparative proteoinformatics revealed the essentials of SDS impact on HaCaT keratinocytes

Timur Shkrigunov[1,*], Yulia Kisrieva[2], Natalia Samenkova[2], Olesya Larina[2], Victor Zgoda[3], Alexander Rusanov[4], Daniil Romashin[4], Natalia Luzgina[4], Irina Karuzina[2], Andrey Lisitsa[1], Natalia Petushkova[2]

[1] Center of Scientific and Practical Education, Institute of Biomedical Chemistry, Moscow 119121, Russia

[2] Laboratory of Microsomal Oxidation, Institute of Biomedical Chemistry, Moscow 119121, Russia

[3] Laboratory of Systems Biology, Institute of Biomedical Chemistry, Moscow 119121, Russia

[4] Laboratory of Precision BioSystems, Institute of Biomedical Chemistry, Moscow 119121, Russia

[*] Correspondence

Timur Shkrigunov, Institute of Biomedical Chemistry, Moscow 119121, Russia

Email: shkrigunov05@mail.ru

Supporting Information

Reagents

Tris(hydroxymethyl)aminomethane (Tris), NaCl, protease inhibitor E64, bicarbonate ammonium, acetonitrile, iodoacetamide, 4-vinylpyridine, trifluoroacetic acid, bicinchoninic acid, and bovine serum albumin (BSA) were purchased from Sigma-Aldrich (St. Louis, MO, USA). Sodium dodecyl sulfate (SDS), dithiothreitol (DDT), and Pierce MS Grade Trypsin Protease were from Thermo Scientific Promega (Madison, WI, USA). Disodium ethylenediaminetetraacetate dihydrate (EDTA) was from BioChemica Applichem (Darmstadt, Germany). Phenylmethylsulfonyl fluoride (PMSF) was from Thermo Scientific (Waltham, MA, USA). Coomassie Brilliant Blue R-250 and glycerol were from Acros Organics (Geel, Belgium). Tris(2-carboxyethyl)phosphine hydrochloride (TCEP) was from Hampton Research (Aliso Viejo, CA, USA).

Cell cultivation and SDS exposure

Cell cultivation and SDS exposure were carried out as described earlier [1]. HaCaT cells were seeded in T75 tissue culture flasks and cultured in DMEM supplemented with 10% FBS and antibiotics (100 U/mL penicillin and 100 g/mL streptomycin). Cells were kept at 37 °C in a humidified 5% CO_2 atmosphere. The medium was changed every other day. After growing to ~60–70% confluence, the cultured cells were divided into two groups: one was control (fresh medium was added), and another was exposed to SDS (25 µg/mL) diluted in complete DMEM. The medium was aspirated 48 hours later, and the cells were washed with PBS, trypsinized with 3 mL of 0.25% trypsin-EDTA per flask, and incubated briefly at 37 °C. The cells were microscopically examined to ensure they were completely detached before being transferred to a centrifuge tube. The cells were centrifuged at 1200 rpm for 5 min before being washed three times in ice-cold PBS. Finally, PBS was aspirated, and the pellet was resuspended in 0.2% SDS-containing buffer (Protocol 1) or ice-cold deionized water (Protocol 2).

1DE-gel concentration and in-gel digestion

Samples were mixed with two volumes of sample buffer (0.5 M Tris-HCl (pH 6.8), 2% SDS, 25% glycerol, 5% 2-mercaptoethanol, 0.5% bromophenol blue) and incubated at 95°C within 4 min. An equal amount of protein (50 µg per lane) was loaded onto three parallel gel lanes. 1DE-gel concentration procedure [2] without fractionating in resolving gel for SDS removal was performed in an upper stacking 4% polyacrylamide gel (2.5 cm)

at 50 V on a Mini-Protean III Cell device (7 × 11 cm) (Bio-Rad, Hercules, CA, USA). The electrophoresis was stopped before the migration of Bromophenol blue into the resolving 12% gel (to the phase boundary, 20 minutes). After concentrating the proteins in the upper gel, the gel was stained with 0.1% Coomassie Brilliant Blue R-250 to obtain a single narrow protein band with a thickness of about 2 mm.

Each gel band was further diced into 1 mm^3 cubes using a scalpel, and in-gel digestion with trypsin was performed according to the standard procedure [3]. Briefly, each band was incubated in destaining buffer (50% acetonitrile (v/v) in 100 mM ammonium bicarbonate, pH = 8.9) for 45–60 min at 50 °C; destaining was repeated twice. Next, each probe was reduced with 45 mM DTT at 56 °C for 60 min, alkylated with 100 mM IAA for 15 min at room temperature in the dark. After dehydration, each was subjected to in-gel proteolysis with trypsin. For this purpose, 6.3 ± 2.0 µL of trypsin solution (25 ng/µL modified trypsin in 50 mM bicarbonate ammonium) was added, depending upon its relative staining, and the mixture was incubated for 1 h at 37 °C. After this, an additional trypsin solution was added, and the mixtures were incubated for 18 h at 37 °C. Then, 15 µL of 0.7% trifluoroacetic acid was added to each gel piece and the samples were incubated for 2 h at room temperature. The mixture of proteolytic peptides from each gel band was used for LC–MS/MS analysis.

In-solution tryptic digestion

The pair of water extracts (175 µg of protein) for each study group, namely control and SDS-exposed HaCaT cells, were in-solution digested in accordance with the protocol described earlier [2]. Protein denaturation and disulfide bond reduction were performed with a solution containing 87 mM DTT and 6.7 mM TCEP in denaturation buffer (12 mM sodium deoxycholate, 2 M thiourea, 2.5 mM EDTA, and 75 mM Tris-HCl, pH = 8.5), and further incubated at 42 °C for 60 min. The reduction solution was added to each sample in a ratio of volume of reduction solution/total protein weight of 1/1 and mixed.

Then, alkylation solution (100 µL of denaturation buffer, 10 µL of 4-vinylpyridine, and 90 µL of N,N-dimethylformamide, pH < 9.0) was added to each sample in a ratio of volume of alkylation solution/volume of sample of 1/12 and mixed thoroughly. The reaction mixture was incubated at 20 °C, for 60 min, in a place inaccessible to daylight.

The digestion buffer containing 100 mM $CaCl_2$ and 42 mM triethylammonium bicarbonate (TEAB, 42 µL) in H_2O (water for UV, HPLC, ACS) was added (up to 100

μL). Trypsin in the ratio 1/100 (trypsin/total protein weight) was added to the sample and then it was incubated in the dark at 44 °C and 50 rpm for 120 min in a GFL Shaking Incubator 3032 (GFL, Burgwedel, Germany). Then, another ratio of trypsin was added, and the solution was incubated in the dark at 37 °C and 50 rpm for an additional 120 min in a GFL Shaking Incubator 3032. The enzymatic digestion was stopped by adding formic acid to a final concentration of 1% and then the sample was centrifuged at room temperature (30 min, 10,000× g).

LC–MS/MS analysis

One microgram of peptides in a volume of 1–4 μL was loaded onto the Acclaim μ-Precolumn (0.5 mm × 3 mm, 5 μm particle size, Thermo Scientific) at a flow rate of 10 μL/min for 4 min in an isocratic mode of Mobile Phase C (2% acetonitrile, 0.1% formic acid). Then, the peptides were separated with high-performance liquid chromatography (HPLC, Ultimate 3000 Nano LC System, Thermo Scientific) in a 15-cm--long C18 column (Acclaim PepMap RSLC inner diameter of 75 μm, Thermo Fisher Scientific). Next, the peptides were eluted with a gradient of buffer B (80% acetonitrile, 0.1% formic acid) at a flow rate of 0.3 μL/min. The total runtime was 90 minutes: this included an initial 4 min of column equilibration to buffer A (0.1% formic acid) and then a gradient from 5 to 35% of buffer B over 65 min and 6 min to reach 99% of buffer B, flushing 10 min with 99% of buffer B and 5 min re-equilibration to buffer A.

MS analysis was performed with a Q Exactive HF-X mass spectrometer (Q Exactive HF-X Hybrid Quadrupole-Orbitrap mass spectrometer, Thermo Fisher Scientific). The capillary temperature was 240 °C, and the voltage at the emitter was 2.1 kV. Mass spectra were acquired at a resolution of 120,000 (MS) in a range of 300−1500 m/z. Tandem mass spectra of fragments were acquired at a resolution of 15,000 (MS/MS) in the range from 100 m/z to m/z value determined by a charge state of the precursor, but no more than 2000 m/z. The maximum integration time was 50 ms and 110 ms for precursor and fragment ions, correspondently. The AGC target for precursor and fragment ions was set to 1106 and 2105, correspondently. An isolation intensity threshold of 50,000 counts was determined for precursor selection, and up to the top 20 precursors were chosen for fragmentation with high-energy collisional dissociation (HCD) at 29 NCE. Precursors with a charge state of +1 and more than +5 were rejected, and all measured precursors were dynamically excluded from triggering a subsequent MS/MS for 20 s.

References

1. Rusanov, A. L., Luzgina, N. G. & Lisitsa, A. V. Sodium dodecyl sulfate cytotoxicity towards HaCaT keratinocytes: comparative analysis of methods for evaluation of cell viability. *Bull. Exp. Biol. Med.* **163(2)**, 284-288; https://doi.org/10.1007/s10517-017-3785-z (2017).

2. Shkrigunov, T. *et al.* Protocol for increasing the sensitivity of MS-based protein detection in human chorionic villi. *Curr. Issues Mol. Biol.* **44(5)**, 2069–2088; https://doi.org/10.3390/cimb44050140 (2022).

3. Shevchenko, A., Wilm, M., Vorm, O. & Mann, M. Mass spectrometric sequencing of proteins silver-stained polyacrylamide gels. *Anal. Chem.* **68**, 850–858; https://doi.org/10.1021/ac950914h (1996).

Suppl. Table 1. The list of proteins identified using SearchGUI in control HaCaT keratinocytes processed by Protocol 1 (0.2% SDS solubilization and 1DE-gel concentration). Corresponds to the data available via ProteomeXchange with identifier PXD035202.

Suppl. Table 2. The list of proteins identified using SearchGUI in SDS-exposed HaCaT keratinocytes processed by Protocol 1 (0.2% SDS solubilization and 1DE-gel concentration). Corresponds to the data available via ProteomeXchange with identifier PXD035202.

Suppl. Table 3. The list of proteins identified using SearchGUI in control HaCaT keratinocytes processed by Protocol 2 (H_2O-based osmotic shock). Corresponds to the data available via ProteomeXchange with identifier PXD035202.

Suppl. Table 4. The list of proteins identified using SearchGUI in SDS-exposed HaCaT keratinocytes processed by Protocol 2 (H_2O-based osmotic shock). Corresponds to the data available via ProteomeXchange with identifier PXD035202.

Suppl. Table 5. The list of keratinocytes-specific proteins identified by ≥ 2 validated unique peptides in HaCaT keratinocytes with/without SDS-exposure processed by Protocols 1 and 2 (0.2% SDS solubilization + 1DE-gel concentration and H_2O-based osmotic shock, correspondingly).

PRIDE Database Submission Details:

Project Name: Proteome profiling of HaCaT keratinocytes

Project accession: PXD035202
